# Reducing background cytokine expression in epithelial cells without serum starvation

**DOI:** 10.1016/j.mex.2014.10.003

**Published:** 2014-10-16

**Authors:** H. Antypas, B. Libberton, K. Melican

**Affiliations:** Swedish Medical Nanoscience Center, Department of Neuroscience, Karolinska Institutet, Retzius väg 8, SE-171 77 Stockholm, Sweden

**Keywords:** FBS, Renal epithelial cells, Growth medium, Inflammation, Cell culture, Cytokines, Serum starvation

## Abstract

Cellular excretion of inflammatory cytokines is an important experimental read-out in a wide range of molecular biology fields. The addition of serum to growth media provides the optimal growing conditions for most cell types. When studying the effect of bacteria and bacterial products on these cells serum starvation is often performed as a standard procedure [1] to avoid unwanted stimulation by the serum components. The full effect of serum starvation on cell behaviour and inflammatory responses is unknown, though it has been suggested to induce various responses that can interfere with experimental results and conclusions [2]. Serum starvation has been shown to cause cells to undergo apoptosis and autophagy [3,4] as well as superoxide production and increasing cell susceptible to inflammatory stimuli [5]. In order to study stimulation of healthy epithelial cells, a new approach was required that limited unwanted stimulation but supported normal cell growth. Analysis of different serum preparations on the background cytokine expression of renal epithelial cells demonstrated conditions in which the background cytokine expression can be reduced without the need to serum starve the cells. Endotoxin content was not found to be the most relevant factor in inducing an inflammatory response in epithelial cells. Charcoal stripped preparations of foetal bovine serum (FBS) produced the lowest background expression of IL-6 and IL-8 without the need for serum starvation.

• Selection of the serum source allows for cytokine expression experiments to be performed without serum starvation.
• Charcoal stripped preparations of FBS produces the lowest background cytokine expression without serum starvation.
• Serum factors other than endotoxin content influence cytokine secretion.

## Graphical abstract

**Figure:**
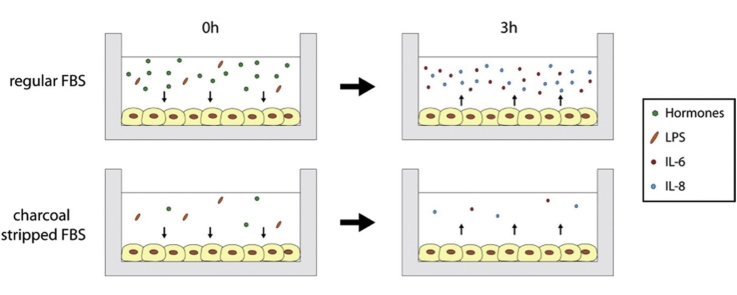

## Method details

### Cell culture media

RPMI 1640 supplemented with 10% FBS (Sigma–Aldrich, Lot 111M3397)

Media tested for inflammation:

**Medium 1**: CO_2_-independent medium (Gibco), 1% GlutaMAX-I (Gibco).
**Medium 2**: CO_2_-independent medium (Gibco), 10% FBS (Sigma–Aldrich, Lot 111M3397, endotoxin = 0.25 EU/ml), 1% GlutaMAX-I (Gibco).
**Medium 3**: CO_2_-independent medium (Gibco), 10% Performance Plus FBS, endotoxin = 0.3 EU/ml (Lot 1399444, Gibco), 1% GlutaMAX-I (Gibco).
**Medium 4**: CO_2_-independent medium (Gibco), 10% FBS Charcoal Stripped (Lot# 1462078, endotoxin = 0.3 EU/ml, Gibco), 1% GlutaMAX-I (Gibco).

### Cell culture

2 × 10^4^ A498 cells (human renal cell carcinoma) were seeded in a 96 well plate (Tissue culture treated, Flat bottom, Sarstedt) and incubated in RPMI 1640 (10%FBS, 1% Gmax) overnight at 37 °C, 95% humidity, 5% CO_2_ to reach confluence.

### Immune response assay

RPMI 1640 was aspirated from the wells and cells were gently washed twice with PBS. 350 μl of media no 1–4 was added. Cells were then incubated at 37 °C, 95% humidity. Supernatants were collected at 0 h and 3 h and stored at −20 °C.

### Cytokine analysis

Cell supernatants were analysed using the Cytometric Bead Array System (BD, Bioscience, Sweden) for IL-6 and IL-8 following the manufacturers procedure. Samples were analysed using a BD FACSCanto II flow cytometer (BD Bioscience, Sweden).

### Viability assay

2 × 10^4^ A498 cells were seeded in a 96-well plate as described above. After overnight incubation, RPMI 1640 medium was removed and cells were gently washed twice with PBS. 100 μl of media 1–4 were added in triplicates. Cells were then incubated at 37 °C, 95% humidity for 3 h. Subsequently, the LIVE/DEAD^®^ Viability/Cytotoxicity Kit (Molecular Probes^®^, Invitrogen, Sweden) was performed according to the manufacturer's instructions. Briefly, cells were incubated with 5 μM ethidium homodimer-1 and 0.5 μM Calcein for 20 min at 37 °C. As a positive control, cells were treated with saponin 0.2% for 10 min prior to LIVE/DEAD stain application, which corresponded to 100% death. Wells with only media 1–4 and LIVE/DEAD stain were used to subtract background fluorescence from the media. To determine viability, cells were excited at 494 nm and fluorescence was detected at 517 nm in a microplate reader (TECAN, Switzerland) to estimate % dead cells or they were observed by epi-fluorescence microscopy (Nikon, Sweden) using a gfp and cy3 filter.

### Data analysis

Data were analysed using the FCAP Array software Version 3.0 (BD Biosciences, Sweden). Final graphs and statistical analysis were performed using Prism 6 (GraphPad Software, USA). Statistical significance was determined using Welch's *t*-test. Statistical significance was determined to be *P* ≤ 0.05.

## Results

**Fig. 1:**
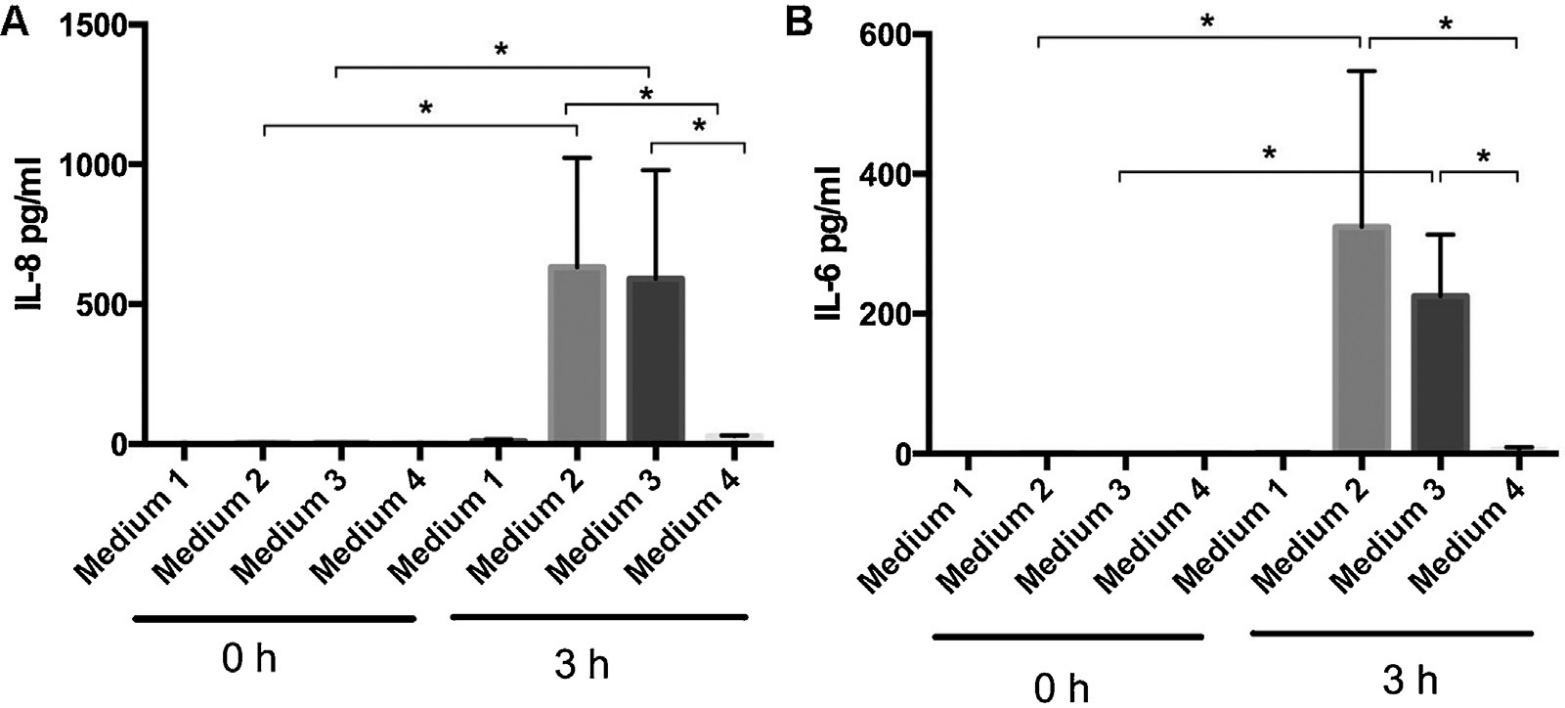
IL-8 and IL-6 expression from A498 renal epithelial cells after 0 h and 3 h incubation in CO_2_ independent media containing the noted FBS preparations. Columns denote median and standard deviation of 3 independent experiments. *P* ≤ 0.05 as determined with Welch's *t*-test.

**Fig. 2:**
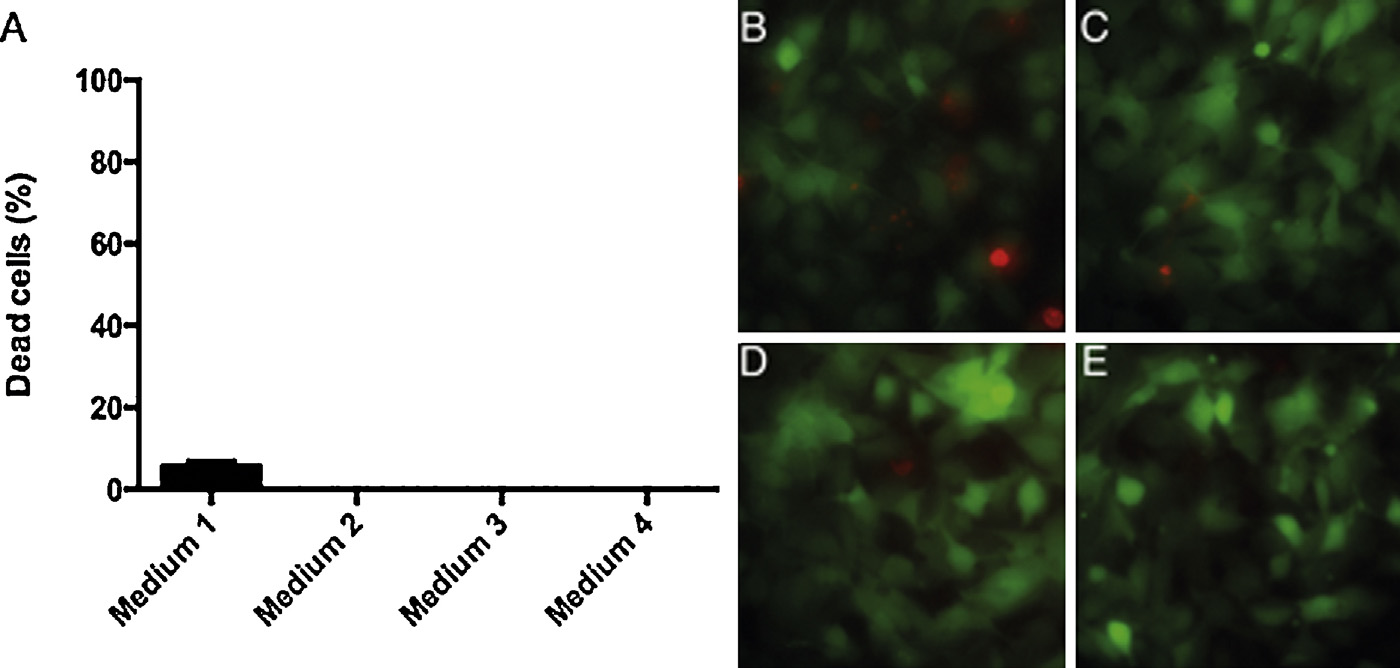
Cell viability after 4 h incubation in CO_2_ – independent medium containing different FBS preparations. (A) Percentage of dead cells stained with ethidium homodimer-1 determined in a microplate reader. (B–E) Live and dead cells stained with calcein and ethidium homodimer-1 and observed with fluorescence microscopy, 20× magnification. B: Medium 1, C: Medium 2, D: Medium 3, E: Medium 4.

Cells cultivated in media containing various FBS preparations demonstrated different levels of inflammatory responses as measured by IL-6 and IL-8 expression (Fig. 1). FBS containing >0.25 EU/ml of endotoxin and Performance Plus FBS which contains less than 0.30 EU/ml of endotoxin induced significant levels of IL-6 and IL-8 expression compared to serum starved cells. Cells incubated in media with FBS which had been charcoal stripped did not express significant levels of IL-6 or IL-8. Cells incubated with charcoal stripped FBS have comparable viability to other FBS preparations, while serum starvation led to an increased percentage of cell death (Fig. 2). This data indicates that this charcoal stripped serum can be used as a media additive in experiments to study the inflammatory response without the need to serum starve the cells.
